# Patterns of Ambulatory Blood Pressure in Normotensive Patients With Type 2 Diabetes Mellitus Attending a Tertiary Care Hospital in South India

**DOI:** 10.7759/cureus.87234

**Published:** 2025-07-03

**Authors:** Noynika Shukla, Tharuni Latha A, Manjunath P R, Sanjana J M, Santhosh Panju, Kavya B K

**Affiliations:** 1 Endocrinology, Diabetes and Metabolism, Ramaiah Medical College and Hospital, Bengaluru, IND; 2 Internal Medicine, Ramaiah Medical College, Bengaluru, IND; 3 Endocrinology, Ramaiah Medical College, Bengaluru, IND; 4 Endocrinology and Metabolism, Ramaiah Medical College, Bengaluru, IND; 5 Endocrinology, Ramaiah Medical College and Hospital, Bengaluru, IND

**Keywords:** abpm, dippers, masked hypertension, non dippers, systemic hypertension, type 2 diabetes mellitus

## Abstract

Systemic hypertension (HTN) is one of the most common comorbidities in diabetes mellitus (DM). Diabetic patients with HTN have a several-fold increased risk of cardiovascular disease (CVD) as compared to normotensive nondiabetic controls. Routine office blood pressure (BP) measurement does not help diagnose hypertensive phenotypes such as masked hypertension, white coat hypertension, etc. Hence, ambulatory blood pressure monitoring (ABPM) is a valuable tool for diagnosing these conditions, as it can help identify adverse cardiovascular complications.

Aims: To study the patterns of ambulatory blood pressure profile in normotensive patients with type 2 DM.

Methodology: This was a cross-sectional study conducted among all patients aged 18 years and above who attended the endocrinology outpatient department of a tertiary care hospital.

Results: The total number of participants in the study was 57, with a mean age of 55.26 years (SD 13.52) among patients. Males comprised the majority, at 32 (56.1%). Masked hypertension was found in 22 (38.6%) patients who had normal blood pressure on routine office BP measurements. Among the masked hypertensive group, 10 (45.5%) patients were dippers, nine (40.9%) were non-dippers, and the rest were reverse dippers. Of those with masked hypertension, 4.54% (n=1) had isolated daytime hypertension, 31.8% (n=7) had isolated nocturnal hypertension, and 63.63% (n=14) had both. Age was the factor that influenced masked hypertension in the study.

Conclusions: The present study underscores the need to incorporate routine utilization of ambulatory blood pressure monitoring in patients with type 2 DM for early identification of hypertension. Solely depending upon office blood pressure monitoring might not be sufficient to detect the hypertensive phenotypes in patients with type 2 DM.

## Introduction

Diabetes is characterized by hyperglycemia resulting from impairment in insulin secretion or action, or both [1]. The burden of diabetes is high and increasing universally and in economically developing nations like India, mainly fueled by the heightened prevalence of overweight/obesity and unhealthy lifestyle practices; the estimates in 2019 showed that 77 million individuals lived with diabetes in India, which is expected to increase to 134 million by 2045 [2]. The chronic hyperglycemia in diabetes is related to long-term complications, dysfunction, and failure of different organs, mainly affecting the eyes, kidneys, nerves, heart, and blood vessels [3]. Hypertension (HTN) is seen in one out of every two patients with diabetes. Diabetic patients with HTN have a fourfold increased risk of cardiovascular disease (CVD) as compared to normotensive, nondiabetic controls [1]. It is a substantial public health problem in India. Hypertension was responsible for 53.8%, 55.7%, and 54.3% of deaths due to coronary heart disease, stroke and chronic kidney disease, respectively, in India in 2016 [4]. Thus, the diagnosis and control of HTN have emerged as the most important intervention in this population. Blood pressure (BP) is a fluctuating phenomenon that has historically been quantified exclusively by static measurements in a physician's office. It continues to be the standard investigation for the diagnosis of HTN and its treatment in all patients. The variability of BP values when the subject's measurement was taken in a medical environment using a sphygmomanometer led to the development of out-of-office BP measurement techniques [5].

The usual measurement method of office blood pressure will not be able to identify masked and white-coat hypertension. Diagnosis and stratification of hypertensive phenotypes are not possible. Recent studies showed that cardiovascular complications are more closely associated with 24-hour and nighttime BP than with office BP. International and national guidelines, having carefully examined the evidence on which method of blood pressure measurement is best - whether office BP, home BP, or ambulatory blood pressure monitoring (ABPM) - have unanimously recommended ABPM as the "gold standard" tool for blood pressure measurement [6]. ABPM is even more relevant for the management of HTN in diabetic patients since HTN is a significant risk factor for CVD in these patients. Diabetic patients are more likely to lose the normal physiological evening dip in blood pressure, known as non-dippers, which is an early phenomenon. Routine office BP measurements cannot recognize these non-dippers. The advantage of ABPM is that it can identify and classify different hypertension phenotypes based on dipping status, such as dippers, non-dippers, reverse dippers, and extreme dippers [7]. The study included a normotensive population to understand the ABPM patterns better without the confounding effect of the drugs and duration of hypertension. Hence, the current study is done to understand the ABPM patterns in patients with type 2 diabetes mellitus (T2DM) for early identification of at-risk individuals. 

## Materials and methods

Methodology

This cross-sectional study was conducted over two months at the Department of Endocrinology. The study was conducted in accordance with the ethical standards established by the institutional ethics committee and the Declaration of Helsinki (1975, as revised in 2000) [8]. Approval for the study was obtained from the Ramaiah Medical College Ethics Committee (approval MSRMC/EC/SP-01/06-2023). Written informed consent was obtained from all participants. All participants were assured of confidentiality, and no personal data (such as names, initials, or hospital numbers) was recorded or disclosed. Participants were informed about their right to withdraw from the study at any stage without affecting their medical care.

Selection and description of participants

Patients with T2DM aged 18 years or above and normal blood pressure based on office blood pressure measurement were included. Patients with existing hypertension, autoimmune disorders, malignancies, pregnancy, existing cardiac, renal and neurological issues were excluded from the study.

Procedure

Informed Consent and Screening

After obtaining written informed consent, participants visiting the Endocrinology Outpatient Department (OPD) were screened as per the inclusion and exclusion criteria. A detailed medical history was obtained, followed by a comprehensive physical examination. Anthropometric measurements were taken with standardized techniques to measure height and weight. BMI was calculated using the weight (kg) / height (m²) formula.

Sample Collection

After an overnight fast of eight to 12 hours, 3 ml of serum and 2 ml of whole blood were collected via phlebotomy under aseptic conditions from each participant. Pre-analytical processing was followed by the analysis of hemoglobin A1C (HbA1C) using the high-performance liquid chromatography (HPLC) method on the Bio-Rad Variant Turbo Analyzer (Hercules, CA, USA). Lipid profiles were analyzed using enzymatic colorimetric methods on the Ortho Clinical Diagnostics VITROS 5600 analyzer (Raritan, NJ, USA).

Blood Pressure Measurement

Office BP was measured according to a standardized procedure using a manual aneroid sphygmomanometer. The mean of the three readings taken one minute apart after five minutes of rest is given before taking the first reading. The patients were subjected to ABPM within 24 hours of their office blood pressure measurement. Ambulatory blood pressure monitoring was performed using a validated, automated oscillometric device (Microlife WatchBP O3 Ambulatory Blood Pressure Monitor (Widnau, Switzerland)), programmed to record blood pressure at 30-minute intervals during the day and 60-minute intervals during the night. The daytime interval was set between 6:00 am and 10:00 pm, and the nighttime interval was set between 10:00 pm and 6:00 am. For analysis, the mean of all valid readings was used. Valid measurements had to fulfil pre-specified quality criteria, including successfully recording at least 70% of programmed measurements, corresponding to 20 daytime and seven nighttime readings, during the 24-hour recording period. Reports were generated on all patients in a standard manner, and after performing ABPM, hypertensive phenotypes were categorized.

Blood pressure definition

Office BP of >130/80mmHg was considered hypertension, which is in accordance with American Heart Association (AHA) guidelines. ABPM value of 24-hour average BP >/=130/80mmHg and average daytime BP >/=125/75mmHg and/or average nighttime BP>/= 110/65mmHg was considered hypertension as ABPM results. Various hypertensive phenotypes based on office blood pressure and ABPM are mentioned in Table 1. 

**Table 1:** Hypertensive Phenotype Terminology Based on Office BP and ABPM BP: blood pressure, ABPM: ambulatory blood pressure monitoring, HTN: hypertension

Parameters	Office BP	ABPM
Normotension	Normal	Normal
Masked HTN	Normal	Elevated

Hypertension subcategories according to ABPM readings are described in Table 2. 

**Table 2:** Subcategories Based on ABPM BP: blood pressure, ABPM: ambulatory blood pressure monitoring, HTN: hypertension

Parameters	Office BP	Overall ABPM	Daytime	Nocturnal
Isolated nocturnal hypertension	Normal	Normal/elevated	Normal	Elevated
Isolated daytime HTN	Normal	Normal/elevated	Elevated	Normal
Daytime and nocturnal HTN	Normal	Elevated	Elevated	Elevated

Dippers are those patients in whom the blood pressure reduces by about 10 - 20% at night compared to daytime readings. Non-dippers are those in whom the nocturnal drop in BP is less than 10%. Reverse dippers are the ones in whom there can actually be an increase in the nighttime BP compared to daytime BP. Hypertension subtypes based on diurnal index are mentioned in Table 3. 

**Table 3:** Classification of Dippers Based on Diurnal Index

Sl no	Pattern	Diurnal index
1	Normal dipper	11-20%
2	Non-dipper	1-10%
3	Reverse dipper	<0
4	Extreme dipper	21 – 30%

Statistical analysis

Sample Size

According to the literature review, a study by Bhat TA et al. found that masked hypertension was present in 53% of normotensive patients with type 2 diabetes mellitus. The present research expects similar results with a 95% confidence level and 13% absolute precision, and the study requires a minimum of 57 participants. Descriptive statistics of masked HTN and isolated nocturnal dipping were analyzed and summarized in percentage. Data was entered into an Excel (Microsoft, Redmond, WA, USA) data sheet and was analyzed using SPSS version 22 (IBM Corp., Armonk, NY, USA) software. Categorical data was represented in the form of frequencies and proportions. The chi-square test or Fisher's exact test (for 2x2 tables only) was used for qualitative data. Continuous data was represented as mean and standard deviation. Student's t-test was used as a test of significance to identify the mean difference between two quantitative variables.

Graphical Representation of Data

Microsoft Excel and Microsoft Word were used to create various graphs. A p-value of <0.05 was considered statistically significant after assuming all the rules of statistical tests.

## Results

The mean age of patients in this study was 55.26 years (SD 13.52), with an average BMI of 24.86 kg/m² (SD 3.38), and HbA1c of 8.33% (SD 1.27) (Table 4).

**Table 4:** Descriptive Statistics of the Study Population HbA1C: hemoglobin A1C, HDL: high-density lipoprotein, LDL: low-density lipoprotein

Parameter	Minimum	Maximum	Mean	Std. Deviation
Age (years)	20	79	55.26	13.515
BMI (kg/m2)	16.7573	31.3213	24.861336	3.3769391
HbA1c (%)	6.4000	12.8000	8.333333	1.2666510
S. Creatinine (mg/dl)	0.50	1.00	0.78	0.12
Cholesterol (mg/dl)	124	226	173.74	25.689
Triglyceride (mg/dl)	100	247	133.81	27.264
HDL (mg/dl)	19	57	39.79	8.752
LDL (mg/dl)	59	158	101.23	23.705

Males constituted a majority in our study at 32 (56.1%), and females were at 25 (43.9%) among the total 57 participants (Table 5).

**Table 5:** Sex Distribution

Sex	Frequency	Percent
Female	25	43.9
Male	32	56.1
Total	57	100.0

Hypertensive phenotypes based on office blood pressure and ABPM measurements consisted of normotensive individuals at 61.4% (n=35), while masked hypertension was found in 38.6% (n=22) of patients (Table 6).

**Table 6:** Hypertension Phenotype Classification

Phenotype	Frequency	Percent
Masked Hypertension	22	38.6
Normotensive	35	61.4
Total	57	100.0

Among the masked hypertensive group, 22 patients were classified accordingly based on dipping status: 10 (45.5%) patients were dippers, nine (40.9%) belonged to non-dippers, and the rest, around three (13.6%), were reverse dippers in the current study (Table 7).

**Table 7:** Dipping Status of the Masked Hypertensive Patients

Dipping Status	Frequency	Percent
Dipper	10	45.5
Non Dipper	9	40.9
Reverse dipper	3	13.6
Total	22	100.0

Hypertensive subcategories in those with masked hypertension were 4.54% (n=1) had isolated daytime hypertension, 31.8% (n=7) had isolated nocturnal hypertension, and 63.63% (n=14) had both.

The age was significantly higher in the masked hypertensives group (60 years vs. 52 years, p = 0.021) compared to the normotensives. Other parameters, such as BMI and HbA1c, showed no significant differences, likely due to the small sample size (Table 8).

**Table 8:** Comparison of Parameters by Hypertension Classification HbA1C: hemoglobin A1C, HDL: high-density lipoprotein, LDL: low-density lipoprotein, HTN: hypertension

	Normotensive		Masked HTN		p value
	Mean	SD	Mean	SD	
Age (years)	52	14	60	12	0.021
BMI (kg/m2)	25.2014	3.1066	24.3203	3.7795	0.342
HbA1c (%)	8.1343	1.1956	8.6500	1.3391	0.136
Cholesterol (mg/dl)	174	27	173	25	0.841
Triglyceride (mg/dl)	132	25	137	31	0.507
HDL (mg/dl)	40	10	40	7	0.768
LDL (mg/dl)	100	23	103	26	0.717

## Discussion

The study provides valuable insights into the distribution of various parameters related to age, gender, dipping patterns in systolic and diastolic blood pressure, classification of hypertension, and descriptive statistics of the study population. Additionally, comparisons were made between normotensive and masked hypertensive groups to assess differences in clinical characteristics and diurnal index.

The study population consisted of individuals across diverse age groups, predominantly middle-aged and older individuals. The distribution of subjects by age showed a relatively even spread, with a mean age of 55.26 years (±13.52). This distribution reflects the higher prevalence of T2DM and HTN among older age groups, consistent with epidemiological trends [9]. Regarding gender distribution, males slightly outnumbered females, aligning with the gender-specific differences in the prevalence of T2DM and HTN reported in previous studies [10].

A notable finding of the study was the high prevalence of masked hypertension (38.6%) among normotensive individuals with T2DM, which was in accordance with the study by Leitão et al [11]. The prevalence of masked hypertension in a study, "The ABPM India study", conducted by Kaul et al. [12] with a large number of participants from the general population, which was 23%, lower than our study on diabetic subjects. Masked hypertension was identified in a substantial proportion of study participants in our study. Among masked hypertensive subjects, various phenotypes were observed out of which one patient had isolated daytime hypertension (4.54%), seven patients had isolated nocturnal hypertension and 14 patients had a combination of both, which was higher than in a study conducted by Bhat et al. [1].

In our study based on the diurnal index, 10 (45.5%) patients exhibited a dipping pattern, and nine (40.9%) patients had a non-dipping pattern. Non-dipping hypertension is associated with heightened cardiovascular risk, underscoring the importance of monitoring diurnal blood pressure variations in patients with T2DM [12]. The presence of non-dipping patterns in the study participants underscores the need for targeted interventions to optimize nocturnal blood pressure control and mitigate cardiovascular risk [3]. This highlights the complexity of hypertension diagnosis and the limitations of relying solely on office blood pressure recordings in patients with T2DM [4]. Ambulatory blood pressure monitoring emerged as a valuable tool for identifying masked hypertension and optimizing treatment decisions in this population. In our study, there were numerically more patients exhibiting dipping status than dippers, probably because of the small study sample.

We also assessed the factors influencing the hypertensive phenotypes both clinical and biochemical parameters like age, gender, BMI, HBA1c and lipid profile of the patients. Out of the factors, only age had a significant association, i.e., elderly patients had higher chances of having masked hypertension in our study. However, we did not find an association between other factors, such as sex, BMI, or HbA1c, as predictors of masked hypertension, as reported in previous studies, likely due to the short study duration and small sample size.

These findings highlight the importance of comprehensive risk assessment and tailored management strategies to mitigate cardiovascular risk in this high-risk population [13]. The mechanism of loss of nocturnal dip is secondary to lower sympathetic activity during sleep leading to decreased heart rate, reduced cardiac activity and loss of vasoconstriction of blood vessels. Other reasons are a decrease in the renin-angiotensin-aldosterone system, altered circadian rhythm due to autonomic dysfunction. The non-dipping pattern is seen in resistant hypertension, secondary hypertension, sleep apnea, and diabetic patients. The loss of this phenomenon or reverse dipping pattern may not have significant clinical symptoms but is associated with several subclinical organ systems, leading to long-term complications [13]. The relationship between masked hypertension and long-term complications of not only cardiovascular but also other systems is well known in the literature [14-16].

Several studies done on hypertensive patients concluded saying more than half of the study population had a non-dipping pattern and were associated with higher left ventricular mass, carotid intima media thickness and microalbuminuria [17,18]. Non-dipping pattern is also associated with retinopathy and nephropathy, highlighting the importance of identifying this pattern which helps to modify the antihypertensive therapy to reduce the adverse cardiovascular impact [18].

Unfortunately, routine office blood pressure measurements do not identify either non-dippers or reverse dippers; ABPM is a unique tool that helps us diagnose these two patterns. A reverse dipping pattern is again an important parameter that can be diagnosed by ABPM alone. Although the data about its relation with subclinical organ damage is scant, very few studies support the association [19]. 

Strengths and limitations

The strength of this study was the utility of ABPM to identify the subclinical phase of hypertension, which has an impact on cardiovascular morbidity and mortality. There is very limited Indian data on the utility of ABPM in normotensive diabetic patients. Limitations were a small sample size and a short duration of study.

## Conclusions

Our study highlights the importance of ABPM in diagnosing the missed cases of masked hypertension and its patterns in patients living with type 2 diabetes. Early and timely identification of masked hypertension helps in intensifying the follow-up and more frequent screening for complications. Identification of non-dippers will benefit from chronotherapy, i.e., appropriate timing of medications. ABPM is a unique screening tool for identifying hypertensive phenotypes and their patterns in type 2 diabetic patients, which should be used judiciously in our practice.

## References

[1] Bhat TA, Mir MR, Naqati S, Naik M, Naqash M (2022). Hypertensive phenotypes and pattern of ambulatory blood pressure in patients of diabetes mellitus of Kashmir Valley. Indian J Endocrinol Metab.

[2] Verdecchia P, Schillaci G, Porcellati C (1991). Dippers versus non-dippers. J Hypertens Suppl.

[3] Salles GF, Reboldi G, Fagard RH (2016). Prognostic effect of the nocturnal blood pressure fall in hypertensive patients: the Ambulatory Blood Pressure Collaboration in Patients With Hypertension (ABC-H) meta-analysis. Hypertension.

[4] Pierdomenico SD, Lapenna D, Bucci A (2005). Cardiovascular outcome in treated hypertensive patients with responder, masked, false resistant, and true resistant hypertension. Am J Hypertens.

[5] (2022). Cardiovascular disease and risk management: standards of medical care in diabetes-2022. Diabetes Care.

[6] Whelton PK, Carey RM (2017). The 2017 clinical practice guideline for high blood pressure. JAMA.

[7] Li Y, Yang L, Wang L (2017). Burden of hypertension in China: a nationally representative survey of 174,621 adults. Int J Cardiol.

[8] (2000). World Medical Association Declaration of Helsinki: ethical principles for medical research involving human subjects (2000). JAMA.

[9] Almdal T, Scharling H, Jensen JS, Vestergaard H (2004). The independent effect of type 2 diabetes mellitus on ischemic heart disease, stroke, and death: a population-based study of 13,000 men and women with 20 years of follow-up. Arch Intern Med.

[10] Liu X, Gu W, Li Z, Lei H, Li G, Huang W (2017). Hypertension prevalence, awareness, treatment, control, and associated factors in Southwest China: an update. J Hypertens.

[11] Gorostidi M, de la Sierra A, González-Albarrán O (2011). Abnormalities in ambulatory blood pressure monitoring in hypertensive patients with diabetes. Hypertens Res.

[12] Kaul U, Arambam P, Rao S (2020). Usefulness of ambulatory blood pressure measurement for hypertension management in India: the India ABPM study. J Hum Hypertens.

[13] de la Sierra A, Redon J, Banegas JR (2009). Prevalence and factors associated with circadian blood pressure patterns in hypertensive patients. Hypertension.

[14] Tientcheu D, Ayers C, Das SR (2015). Target organ complications and cardiovascular events associated with masked hypertension and white coat hypertension: analysis from the Dallas Heart Study. J Am Coll Cardiol.

[15] Ingelsson E, Björklund-Bodegård K, Lind L, Arnlöv J, Sundström J (2006). Diurnal blood pressure pattern and risk of congestive heart failure. JAMA.

[16] Kumar SK (2018). Clinical profile and target organ damage in non-dipper hypertensives: a hospital-based study. J Indian Coll Cardiol.

[17] Mishra A, Kumar R, Saran R (2017). Non-dipping blood pressure pattern and its association with target organ damage in essential hypertension. J Clin Diagn Res.

[18] Ghosh A, Ray BK, Ghosal A (2021). Study of nocturnal blood pressure dipping pattern and its correlation with diabetic microvascular complications in hypertensive type 2 diabetes mellitus patients. Indian Heart J.

[19] Cuspidi C, Sala C, Tadic M, Gherbesi E, De Giorgi A, Grassi G, Mancia G (2017). Clinical and prognostic significance of a reverse dipping pattern on ambulatory monitoring: an updated review. J Clin Hypertens (Greenwich).

